# *Nkx2-5* and Sarcospan genetically interact in the development of the muscular ventricular septum of the heart

**DOI:** 10.1038/srep46438

**Published:** 2017-04-13

**Authors:** Adam A. Panzer, Suk D. Regmi, DePorres Cormier, Megan T. Danzo, Iuan-bor D. Chen, Julia B. Winston, Alayna K. Hutchinson, Diana Salm, Claire E. Schulkey, Rebecca S. Cochran, David B. Wilson, Patrick Y. Jay

**Affiliations:** 1 Department of Pediatrics, Washington University School of Medicine, Box 8208 660 South Euclid Avenue, St. Louis, MO, 63110, USA; 2 Department of Developmental Biology, Washington University School of Medicine, Box 8208 660 South Euclid Avenue, St. Louis, MO, 63110, USA; 3 Department of Genetics, Washington University School of Medicine, Box 8208 660 South Euclid Avenue, St. Louis, MO, 63110, USA.

## Abstract

The muscular ventricular septum separates the flow of oxygenated and de-oxygenated blood in air-breathing vertebrates. Defects within it, termed muscular ventricular septal defects (VSDs), are common, yet less is known about how they arise than rarer heart defects. Mutations of the cardiac transcription factor *NKX2-5* cause cardiac malformations, including muscular VSDs. We describe here a genetic interaction between *Nkx2*-*5* and *Sarcospan (Sspn*) that affects the risk of muscular VSD in mice. *Sspn* encodes a protein in the dystrophin-glycoprotein complex. *Sspn* knockout (*Sspn*^KO^) mice do not have heart defects, but *Nkx2-5*^+/−^/*Sspn*^KO^ mutants have a higher incidence of muscular VSD than *Nkx2*-*5*^+/−^ mice. Myofibers in the ventricular septum follow a stereotypical pattern that is disrupted around a muscular VSD. Subendocardial myofibers normally run in parallel along the left ventricular outflow tract, but in the *Nkx2-5*^+/−^/*Sspn*^KO^ mutant they commonly deviate into the septum even in the absence of a muscular VSD. Thus, *Nkx2-5* and *Sspn* act in a pathway that affects the alignment of myofibers during the development of the ventricular septum. The malalignment may be a consequence of a defect in the coalescence of trabeculae into the developing ventricular septum, which has been hypothesized to be the mechanistic basis of muscular VSDs.

Muscular VSDs are anatomically simple, but their pathogenesis is ironically less well understood than more complex heart defects such as tetralogy of Fallot. Two general hypotheses, extrapolated from observations of normal cardiac development in the chick embryo, were proposed decades ago to explain how a muscular VSD arises. Early observations of cell death suggested that excessive apoptosis could perforate the septum^1^,^2^. Increased myocyte apoptosis is not typically observed in association with abnormal ventricular septal development, however, even if the genetic perturbation is severe^3^. Alternatively, the ventricular septum in chick embryos forms by the coalescence of trabeculae^4^,^5^,^6^. Muscular VSDs were hence proposed to represent the persistence of gaps between trabeculae during their coalescence^5^.

The development of the muscular ventricular septum in mouse models has not been scrutinized as closely as other cardiac structures, but morphologic observations support the role of trabecular coalescence^7^,^8^. Certain mutant phenotypes suggest that the process of coalescence shares genetic pathways with trabecular development. For example, conditional knockout mutations that disrupt Notch signaling cause hypertrabeculation and ventricular noncompaction phenotypes similar to those observed in humans. Investigators have thus focused their attention upon the ventricular myocardium of the free wall, but they have made note of muscular and membranous VSDs^9^,^10^,^11^. Curiously, the ventricular septum in the mutants is not necessarily hypertrabeculated or thin like the compact myocardium of the free wall. Some regions of the ventricular septum can have normal thickness and shape, while gaps elsewhere suggest a local failure of trabeculae to coalesce (cf. Fig. 6 in ref. 8, Fig. 1a in ref. 9, Fig. 4a in ref. 10, and Figs 2 and 7 in ref. 12, which show examples from cardiomyocyte-specific knockouts of *Mib1*^8^,^9^, *Fkbp1a*^10^, and *Scrib* and *Rac1*^12^).

If the conditional knockout mutants above represent extreme examples of how muscular VSDs arise, then subtler signs of abnormal trabecular coalescence may be associated with milder mutations of cardiac developmental genes that cause muscular VSD. Among the genes, mutations of *NKX2-5*, a cardiac transcription factor, cause pleiotropic congenital heart defects^13^,^14^. The anatomic phenotype is determined in part by modifier genes that influence the susceptibility of specific developmental pathways to *Nkx2-5* mutation^14^,^15^. One modifier locus on chromosome 6 for muscular VSDs was previously mapped in a cross between the C57BL/6N and FVB/N inbred strains^15^. The paucity of knowledge regarding how a muscular VSD arises motivated us to evaluate sarcospan, a gene in the locus.

Sarcospan (SSPN) is a transmembrane protein in the dystrophin-glycoprotein complex. The complex links the extracellular matrix to the membrane cytoskeleton of myocytes. *Sspn* is specifically expressed in the heart and skeletal muscle^16^,^17^. Mutations of dystrophin-glycoprotein complex genes cause muscular dystrophy. Mutations are not usually associated with congenital heart disease, although an alpha-dystrobrevin mutation in one family was associated with VSD, hypoplastic left heart, and left ventricular noncompaction^18^. C57BL/6N, which bears the risk allele at the chromosome 6 locus, carries a missense mutation of a highly conserved glutamine at position 213 of SSPN. The amino acid is in the cytoplasmic C-terminal domain (Fig. 1a). *Sspn* knockout (*Sspn*^KO^) mice have normal skeletal muscle function. The null mutant is obtained at the expected Mendelian frequency^19^, so *Sspn* deficiency alone would not be expected to cause serious congenital heart defects.

## Results

### Identification of *Sarcospan* as a candidate modifier gene for muscular VSD in *Nkx2-5* ^+/−^ mice

**Figure 1:**
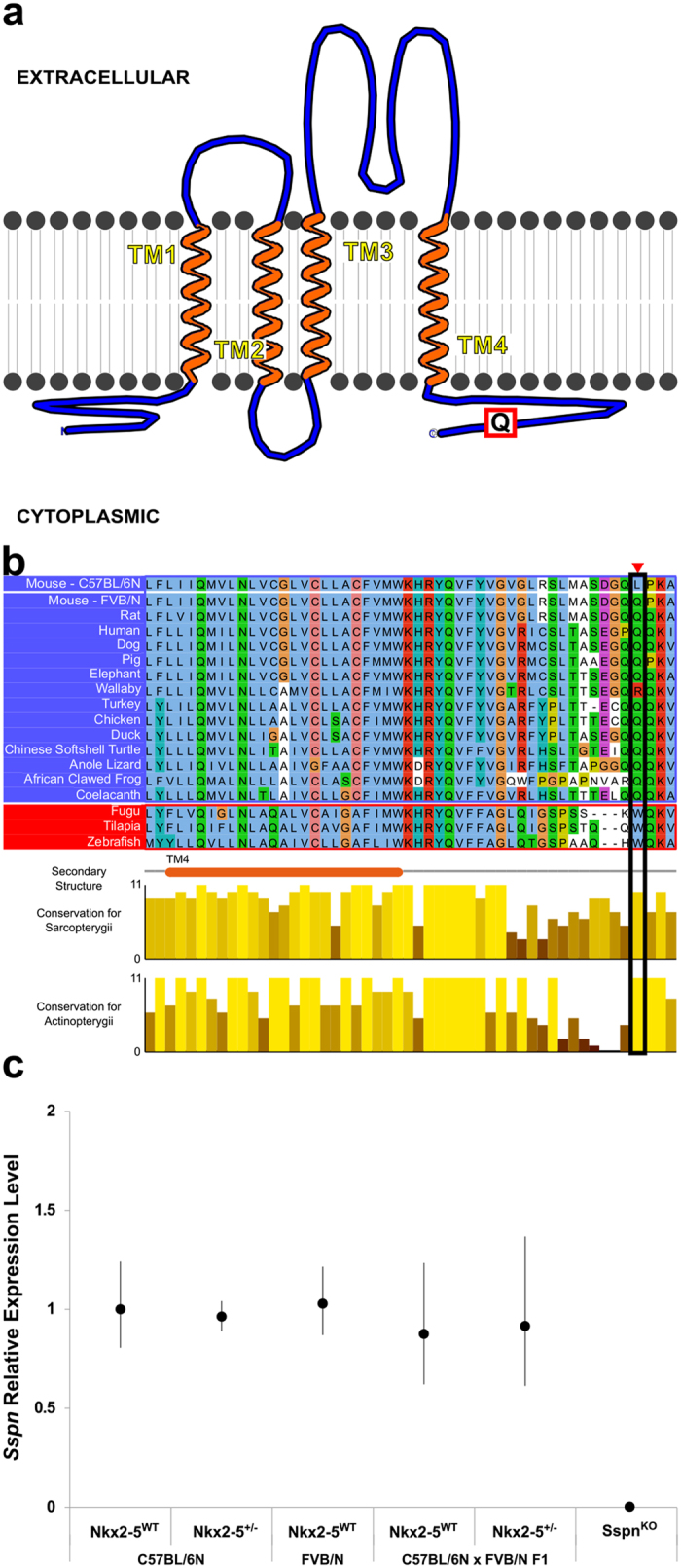
*Sspn* is a candidate modifier gene for muscular VSD susceptibility. (**a**) SSPN is a transmembrane protein in the dystrophin-glycoprotein complex. The highly conserved glutamine (Q) at position 213 of mouse SSPN resides in the C-terminal cytoplasmic domain. (**b**) Q213 is nearly invariant across *Sarcopterygii* species. C57BL/6 carries a missense mutation (Q213L), whereas FVB/N carries the conserved glutamine. The multispecies alignment in the C-terminal portion of the protein is shown for representative *Sarcopterygii* and *Actinopterygii* species. Mouse position 213 is indicated. Amino acid conservation is scored separately for the *Sarcopterygii* and *Actinopterygii*. The maximum score, 11, corresponds to perfect conservation of an amino acid across all species. The full-length sequences of additional species are aligned in Supplementary Fig. 1. (**c**) The expression of *Sspn* mRNA in the heart is the same across the inbred C57BL/6N (N = 3 per genotype), FVB/N (N = 3) and C57BL/6N X FVB/N F1 hybrid backgrounds (N = 6 per genotype) and wild-type and *Nkx2-5*^+/−^ genotypes. No transcript is detected in *Sspn*^KO^ hearts (N = 2). Mean ± S.D. are shown.

We sequenced the *Sspn* alleles carried by the parental strains in our mapping cross. We confirmed that FVB/N carries the conserved glutamine (Q) and C57BL/6N carries a leucine (L) at position 213 (Fig. 1b and Supplementary Fig. 1). The function of the conserved glutamine at position 213, which aligns to position 240 in humans, is unknown, but a survey of human and other vertebrate species suggests that the amino acid was strongly selected at the divergence of lobe- and ray-finned fishes. All 65,777 humans in the 1000 Genomes Project^20^, NHLBI GO Exome Sequencing Project^21^, and the Exome Aggregation Consortium^22^ and nearly all *Sarcopterygii* species, e.g., coelacanth, lungfish and tetrapods, carry a glutamine at the position mutated in C57BL/6 (Fig. 1b and Supplementary Fig. 1). The equivalent Q240L mutation in human *SSPN* has a scaled CADD score of 12.4, placing it in the top 10% of potential variants ranked by predicted deleteriousness^23^. *Actinopterygii*, i.e., ray-finned fishes, all carry a tryptophan (W) at the same position (Fig. 1b and Supplementary Fig. 1). Q240W has a scaled CADD score of 25, placing it in the top 1% of predicted deleterious variants^23^.

Some chromatin immunoprecipitation data suggest that NKX2-5 binds to potential *Sspn* regulatory elements^24^,^25^, but we found no evidence for a regulatory polymorphism that affects expression. *Sspn* mRNA levels are the same in the hearts of C57BL/6N, FVB/N and C57BL/6N X FVB/N F1 hybrid mice. Wild-type and *Nkx2-5*^+/−^ mice also express *Sspn* at the same level (Fig. 1c).

### The *Sspn* null mutation increases the incidence of muscular VSD in *Nkx2-5* ^+/−^ mice

We next investigated whether *Sspn* genetically interacts with *Nkx2-5* in the development of the muscular ventricular septum. Double heterozygotes, i.e., *Nkx2-5*^+/−^/*Sspn*^+/−^, were crossed to each other or *Sspn*^+/−^ single mutants. The *Nkx2-5* null mutation is embryonic lethal^26^,^27^, but all other combinations of *Nkx2-5* and *Sspn* genotypes were recovered at the expected Mendelian frequency in newborn mouse pups (Supplementary Fig. 2). The co-occurrence of *Nkx2-5*^+/−^ and a heterozygous or homozygous *Sspn* knockout mutation does not affect embryonic survival.

**Figure 2:**
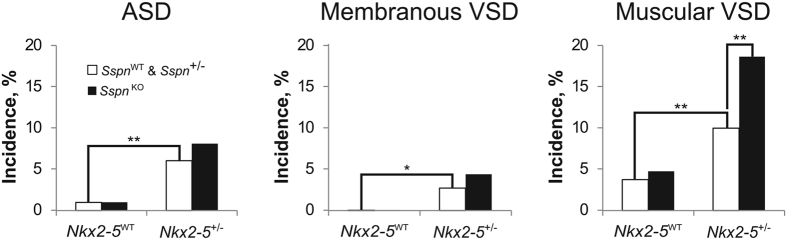
The *Sspn*^KO^ mutation increases the incidence of muscular VSD in *Nkx2-5*^+/−^ mice. (**a**) *Nkx2-5*^+/−^ mice have an increased incidence of ASD and membranous and muscular VSD compared to the *Nkx2-5*^WT^, regardless of the *Sspn* genotype. *Sspn*^WT^ and *Sspn*^+/−^ genotypes are combined because their separate incidences of any defect are the same regardless of *Nkx2-5* genotype. The *Sspn*^KO^ genotype increases the incidence of muscular VSD in the *Nkx2-5*^+/−^ background but not ASD or membranous VSD. The *Sspn*^KO^ mutation alone does cause heart defects. N = 217 *Nkx2-5*^WT^/*Sspn*^WT^ & /*Sspn*^+/−^, 106 *Nkx2-5*^WT^/*Sspn*^KO^, 301 *Nkx2-5*^+/−^/*Sspn*^WT^ & /*Sspn*^+/−^, 161 *Nkx2-5*^+/−^/*Sspn*^KO^. *P < 0.05. **P < 0.01.

The null mutation of *Sspn (Sspn*^KO^) does not cause congenital heart defects in *Nkx2-5* wild-type (*Nkx2-5*^WT^) mice (Fig. 2). The incidences of each type of septal defect among the *Sspn* wild-type (*Sspn*^WT^) and *Sspn*^+/−^ mice in either the *Nkx2-5*^+/−^ or *Nkx2-5*^WT^ groups were the same, so their results were combined for statistical analyses.

As expected, *Nkx2-5*^+/−^ mice have an increased incidence of atrial septal defects (ASDs), membranous VSDs and muscular VSDs compared to the *Nkx2-5*^WT^. The *Sspn*^KO^ genotype does not affect the incidences of ASD or membranous VSD in the *Nkx2-5*^+/−^ mice, but *Nkx2-5*^+/−^/*Sspn*^KO^ double mutants have almost twice the incidence of muscular VSD compared to *Nkx2-5*^+/−^ single mutants (Fig. 2). The results place *Sspn* in a genetic pathway that leads from *Nkx2-5* mutation to a defect in the muscular ventricular septum.

Incidentally, a low incidence of muscular VSD was observed in *Nkx2-5*^WT^ hearts. In a previous study of *Nkx2-5*^WT^ mice in the C57BL/6N strain, which is also the predominant background of the mice in this study, we did not detect the <5% incidence probably because of a smaller sample size^14^. The prior study did note, however, a correlation between muscular VSD incidence in *Nkx2-5*^+/−^ mice and the fraction of C57BL/6N genome in the genetic background of inbred strain crosses with FVB/N and A/J. C57BL/6N may carry genetic polymorphisms that increase the propensity toward muscular VSD in *Nkx2-5*^WT^ hearts as well.

### The *Sspn* null mutation disrupts the organization of myocytes in the ventricular septum of *Nkx2-5* ^+/−^ mice

**Figure 3:**
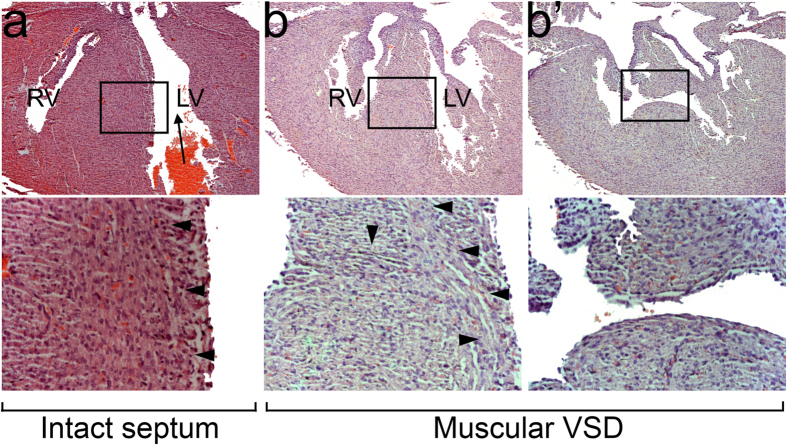
The course of myofibers deviates in and around a muscular VSD. (**a**) In an *Nkx2-5*^+/−^ heart with an intact ventricular septum, the subendocardial fibers on the left side of the septum run parallel to the outflow tract, as indicated by the arrow. (**b**,b’) In an *Nkx2-5*^+/−^ heart with a muscular VSD, the left-sided subendocardial fibers deviate toward the right and split in two directions in the region just ventral to the VSD. One branch continues to the right side. At the level of the VSD, the fibers are generally malaligned. The upper and lower panels are shown at 5x and 20x magnification, respectively. RV, LV, right and left ventricle.

The inspection of thousands of hearts in several projects led us to notice that the myofibers around a muscular VSD veer from their normal trajectories. For example, in a heart with an intact ventricular septum the subendocardial fibers on the left side of the septum tend to run in parallel from the apex to the base (Fig. 3a). In a heart with a muscular VSD, the left-sided, subendocardial fibers veer toward the right in sections that are near the defect (Fig. 3b). Fibers that surround the defect appear even more malaligned (Fig. 3c). The malalignment could either be a simple consequence of myofiber diversion around a defect or a sign of flawed trabecular coalescence, which could be the basis of a muscular VSD. To distinguish the two possibilities, we cut 1 μm, thin sections of plastic-embedded hearts. The method preserves the myofiber architecture better than paraffin embedding, which is more amenable to sectioning large number of hearts quickly.

The orientation of myofibers in the normal heart follows a stereotypical pattern^28^. In the left ventricular free wall, subepicardial fibers run with a major component parallel to the long axis from apex to base, while fibers in the middle layer run circumferentially around the ventricle. The left ventricular free wall in the four genotypic groups – wild type, *Nkx2-5*^+/−^ *and Sspn*^KO^ single mutants, and *Nkx2-5*^+/−^/*Sspn*^KO^ double mutant – showed this pattern (Supplementary Fig. 3).

**Figure 4:**
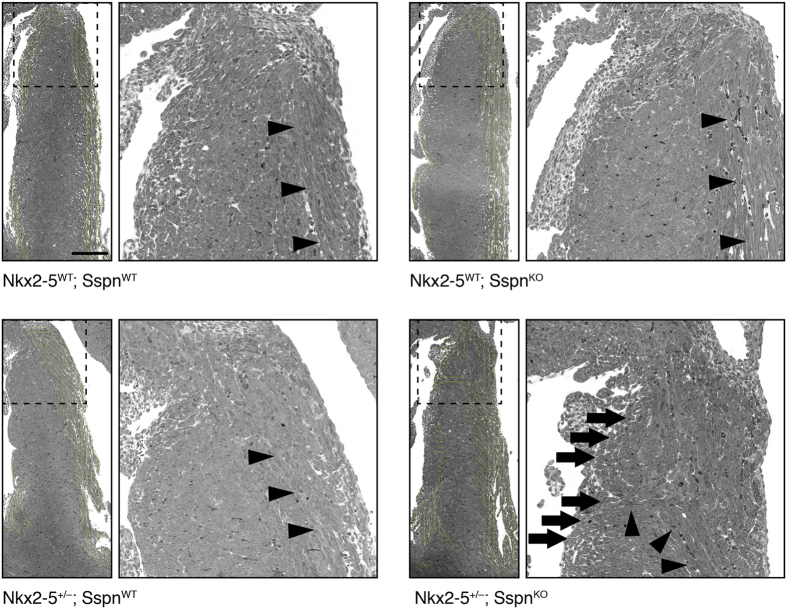
Abnormal course of myofibers in the ventricular septum of *Nkx2-5*^+/−^/*Sspn*^KO^ hearts. The left panel of each image pair shows the ventricular septum of representative wild-type (N = 5), *Sspn*^KO^ (N = 4) and *Nkx2-5*^+/−^ (N = 3) single mutants, and double mutant *Nkx2-5*^+/−^/*Sspn*^KO^ (N = 4) hearts. The yellow vectors indicate the orientation of fibers that run parallel to the plane of section. The right panel shows the orientation of myofibers within the boxed region. Subendocardial myofibers on the left side of the ventricular septum generally run in parallel from the apex to base in every wild-type and single mutant heart examined. In each of the double mutant *Nkx2-5*^+/−^/*Sspn*^KO^ hearts, the left-sided subendocardial fibers take abnormal turns toward the right (arrowheads), sometimes forming whorls. The arrows point to two whorls. None of the hearts examined has a muscular VSD. Scale bar, 200 μm.

In the ventricular septum, the subendocardial fibers run in parallel along the left ventricular outflow tract. Subendocardial fibers on the right ventricular side do not have as consistent a pattern, perhaps owing to the shape of the right ventricle relative to the plane of section. Fibers in the middle layer of the septum run circumferentially. This pattern was observed in the intact ventricular septum of every wildtype and *Nkx2-5*^+/−^ and *Sspn*^KO^ single mutant heart (Fig. 4). In contrast, each of the *Nkx2-5*^+/−^/*Sspn*^KO^ double mutants examined had a subset of subendocardial fibers on the left side of the ventricular septum that deviated toward the right, sometimes forming whorls, even though there was no VSD (Fig. 4 and Supplementary Fig. 4). The incidence of abnormal myofiber organization in the *Nkx2-5*^+/−^/*Sspn*^KO^ double mutant is significantly greater than in the other three genotypic groups (P < 0.001, two-tailed Fisher exact test). An abnormal course of myofibers in the ventricular septum is not simply the consequence of a muscular defect. Collectively, the results indicate that *Nkx2-5* and *Sspn* operate together in a pathway that affects the alignment of myofibers in the ventricular septum.

## Discussion

Dozens of genetic mutations are known to cause muscular VSDs, but how the heart defect forms is poorly understood^29^,^30^. A novel genetic interaction between *Nkx2-5* and *Sspn* offers insight into the mechanism. *NKX2-5* regulates diverse cardiac developmental pathways^13^. Polymorphisms of modifier genes in these pathways influence their susceptibility to *Nkx2-5* haploinsufficiency and the expression of the mutant phenotype^14^. *Sspn* is a gene that resides in a chromosome 6 locus that modifies the risk of muscular VSD in *Nkx2-5*^+/−^ mice^15^. The present data indicate that *Sspn* is an *Nkx2-5* modifier gene, although we cannot state definitively whether it is the gene that underlies the chromosome 6 locus. Complete loss of *Sspn* function does not cause heart defects but does increase the incidence of muscular VSD in combination with an *Nkx2-5* mutation. In addition, myofibers in the ventricular septum of *Nkx2-5*^+/−^/*Sspn*^KO^ double mutants commonly deviate from their normal trajectories. Myofibers similarly deviate around muscular VSDs, as noticed by us and another group^31^. We propose that the malaligned septal myofibers are a sign of a defect in trabecular coalescence that causes muscular VSDs.

The development of the muscular ventricular septum begins with the coalescence of trabeculations in the interventricular groove of the looping heart tube^6^,^7^. Lineage analyses in the mouse demonstrate that left and right ventricular myocytes form their respective sides of the septum. A sharp, left-right boundary delineates their contributions to the septum^32^. Myofibers that run from left to the right side of the ventricular septum appear to violate this boundary. Certain defects of trabecular development could disrupt their coalescence into the ventricular septum, thus causing the malalignment of myofibers or a muscular VSD. For example, cardiomyocyte-specific deletion of *Scrib*, a planar cell polarity gene, causes the stunting of trabeculae. Scrib localizes the GTPase signaling protein Rac1 to the cell membrane. Their interaction plays a critical role in the organization of trabecular myocytes, the apposition of myocytes in the ventricular septum, and the regulation of junctional complex formation at the cell membrane^12^. A quantitative defect in these processes could cause a trabecula to become malaligned if it has no neighboring trabeculae to support its normal coalescence. A muscular VSD could result when malaligned trabeculae do not come into apposition.

Interestingly, there may be subtle, quantitative differences in trabecular developmental processes even between wild-type animals. Fractal analyses indicate that the morphologic pattern of the trabecular network is more complex in the inbred C57BL/6 strain compared to the outbred NIMR:Parkes^8^. This complex, but quantifiable trabecular phenotype may be related to the low incidence of muscular VSDs in *Nkx2-5*^WT^ mice from a predominantly C57BL/6N background. Fractal analyses of trabeculae, as described by Captur *et al*.^8^,^33^, in mutants prone to muscular VSD could help to illuminate patterns of trabecular coalescence during cardiac development and the functional significance of myofiber malalignment.

The molecular and cellular bases of the genetic interaction between *Nkx2-5* and *Sspn* remain to be defined. The hypertrabeculation phenotypes associated with human and mouse mutations indicate that *NKX2-5* plays a role in trabecular development^34^,^35^. The present results indicate that loss of *Sspn* exacerbates a subtle defect associated with *Nkx2-5* haploinsufficiency. In light of what is known about dystrophin-associated complex genes in skeletal muscle and other organisms, one can consider potential physical or biological functions of *Sspn* in ventricular septal development. The dystrophin-glycoprotein complex is best known for its role in maintaining the integrity of the muscle cell membrane and transducing force^36^. Mutations that cause muscular dystrophy are not associated with congenital heart defects, so a purely mechanical function of *Sspn* in ventricular septal development seems less likely. The dystrophin-glycoprotein complex also plays a role in signaling. SSPN promotes Akt signaling during muscle regeneration, and the dystrophin-glycoprotein complex modulates integrin signaling^37^,^38^,^39^. Elegant studies in *Drosophila* clearly demonstrate the functions of the dystrophin-associated complex in regulating planar cell polarity^40^,^41^, BMP and Notch signaling^42^,^43^. All these pathways are all involved in the formation of the ventricular myocardium^11^, and NKX2-5 has been shown to regulate BMP and Notch signaling^44^,^45^.

The function of amino acid Q213 and the effect of the Q213L mutation on *Sspn* function are unknown. The cytoplasmic C-terminal domain of SSPN, which includes Q213, contributes to protein-protein interactions that stabilize the dystrophin-glycoprotein complex^46^. The amino acid could function in this capacity or in a different protein-protein interaction. Interestingly, the conserved glutamine arose in the *Sarcopterygii* lineage in association with the onset of the development of a ventricular septum. The African lungfish, an early *Sarcopterygii* species, has a rudimentary ventricular septum^47^, whereas ray-finned fishes do not. *Sspn* is not essential for the formation of a ventricular septum, but the evolution of the ventricular septum undoubtedly involved the selection of polymorphisms present in the lobe-finned fishes.

Much remains to be learned regarding how mutations of genes like *Nkx2-5* and their interactions with modifier genes cause muscular VSDs. Although muscular VSDs are usually benign, the investigation of their pathogenesis should illuminate mechanisms pertinent to congenital heart disease, cardiomyopathy and cardiac tissue engineering.

## Methods

### Mouse strains

*Sspn* knockout mice were purchased from the Jackson Laboratory. They were predominantly in a C57BL/6 background. The *Sspn* knockout was made in 129S1/Sv x 129X1/SvJ F1 ES cells and backcrossed to C57BL/6 for five generations prior to arrival at Jackson. The *Sspn* wild-type and knockout alleles were genotyped as described^19^. *Nkx2-5*^+/−^ mice were maintained in the C57BL/6N background and genotyped as previously described^14^,^27^. The C57BL/6N and FVB/N inbred strains were purchased from Charles River. The experiments were approved by the animal studies committee at Washington University School of Medicine and performed in accordance with their guidelines.

### Sarcospan sequence analyses

SNPs among inbred mouse strains in *Sspn* were identified through a search of Mouse Genome Informatics (http://www.informatics.jax.org/strains_SNPs.shtml). The sequences of *Sspn* for the C57BL/6N and FVB/N strains in our colony were confirmed by cloning cDNA and sequencing on an Applied Biosystems 3730 DNA analyzer.

SNPs of human *SSPN* were queried on August 17, 2016 in the following databases: 1000 Genomes^20^ (N = 2504 individuals), Exome Aggregation Consortium^22^ (N = 60706), and NHLBI GO Exome Sequencing Project^21^ (N = 6503). The Exome Aggregation Consortium database includes the data of 3936 individuals from the NHLBI GO Exome Sequencing Project.

The reference sequences of *SSPN* from multiple species were downloaded from Ensembl and NCBI on April 15, 2015. Sequences that lacked the C-terminus or the expected stop codon or had large gaps (≥10 amino acids) were excluded from multi-species alignment analysis. The amino acid sequences of 48 species were aligned in Clustal Omega (http://www.ebi.ac.uk/Tools/msa/clustalo/)^48^. The physiochemical conservation scores at each amino acid position was calculated and visualized in Jalview (http://www.jalview.org/)^49^. The transmembrane protein structure of mouse SSPN was predicted with the TMHMM Server v. 2.0 (http://www.cbs.dtu.dk/services/TMHMM/) and drawn with TMRPres2D (http://bioinformatics.biol.uoa.gr/TMRPres2D/)^50^,^51^.

The deleteriousness of an amino acid substitution was estimated on the human *SSPN* sequence by the Combined Annotation-Dependent Depletion (CADD) method^23^. Scaled CADD scores for codon substitutions at human Q240 of *SSPN* were obtained from the CADD website http://cadd.gs.washington.edu/home.

### Quantification of *Sspn* mRNA expression

Total RNA was purified from C57BL/6N and FVB/N hearts using the RNeasy kit (Qiagen). cDNA was synthesized with a SuperScript Vilo cDNA synthesis kit (Invitrogen). Quantitative RT-PCR was performed using SYBR GreenER qPCR SuperMix (Invitrogen) on an Mx3005 P qPCR system (Stratagene). *Sspn* expression levels were normalized to *Gapdh*.

### Phenotyping of congenital heart defects

*Nkx2-5*^+/−^ mice were crossed to *Sspn*^+/−^ or *Sspn*^KO^ mice to generate double heterozygote F1 offspring, i.e., *Nkx2-5*^+/−^/*Sspn*^+/−^, as well as the other genotypes. *Nkx2-5*^+/−^/*Sspn*^+/−^ mice were then crossed to other double heterozygotes or *Sspn*^+/−^ single mutants. The F2 offspring were collected within hours of birth. Their hearts were dissected, fixed in 10% formalin and processed for histology. The hearts were sectioned completely at 6 μm thickness and stained with hematoxylin and eosin. All the sections were examined for congenital heart defects without knowledge of genotype, as previously described^14^.

### Assessment of cardiac myofiber orientation

Newborn mouse hearts were fixed in modified Karnovsky’s fixative (2% paraformaldehyde, 2.5% glutaraldehyde, 0.1 M cacodylate buffer, pH 7.4) and osmium tetroxide. The hearts were embedded in resin. Serial sections through the entire heart were cut at 1 μm thickness in the frontal plane and stained with toluidine blue. Sections were imaged with a Nanozoomer 2.0-HT digital slide scanner (Hamamatsu) at 20x magnification.

The orientation of myofibers was analyzed using ImageJ (http://fiji.sc) and an ImageJ plugin, OrientationJ (http://bigwww.epfl.ch/demo/orientation/)^52^. OrientationJ imposes a square grid on an image. The dominant orientation of pixels within each square is depicted as a vector. The analyses were performed by two individuals blinded to genotype.

## Statistical analyses. 

The incidences of a heart defect in two groups were compared in a 2 × 2 contingency table. Statistical significance was assessed by a chi-squared test.

The incidences of abnormal myofiber orientation were compared in a 2 × 4 contingency table. Statistical significance was assessed by a two-tailed, Fisher exact test.

## Additional Information

**How to cite this article:** Panzer, A. A. *et al. Nkx2-5* and Sarcospan genetically interact in the development of the muscular ventricular septum of the heart. *Sci. Rep.* **7**, 46438; doi: 10.1038/srep46438 (2017).

**Publisher's note:** Springer Nature remains neutral with regard to jurisdictional claims in published maps and institutional affiliations.

## Supplementary Material

Supplementary Information
