# Supplementary material for: Nkx2-5 and Sarcospan genetically interact in the development of the muscular ventricular septum of the heart
Source: Sci Rep. 2017 Apr 13;7:46438. doi: 10.1038/srep46438 (PMC5390293; doi:10.1038/srep46438)
Supplement: Supplementary Information [file srep46438-s1.pdf]

***Nkx2-5* and *Sarcospan* genetically interact in the development of the muscular ventricular septum of the heart**

**Supplementary information**

Adam A. Panzer[1], Suk D. Regmi[1], DePorres Cormier[1], Megan T. Danzo[1], Iuan-bor D. Chen[1], Julia B. Winston[1], Alayna K. Hutchinson[1], Diana Salm[1], Claire E. Schulkey[1], Rebecca Cochran[1], David B. Wilson[1,2], Patrick Y. Jay[1,3,*]

Departments of [1]Pediatrics, [2]Developmental Biology, and [3]Genetics

Washington University School of Medicine

Box 8208

660 South Euclid Avenue

St. Louis MO 63110

*Correspondence to PYJ.

Phone:  314-362-2174

Fax:  314-286-2892

E-mail:  jay_p@kids.wustl.edu

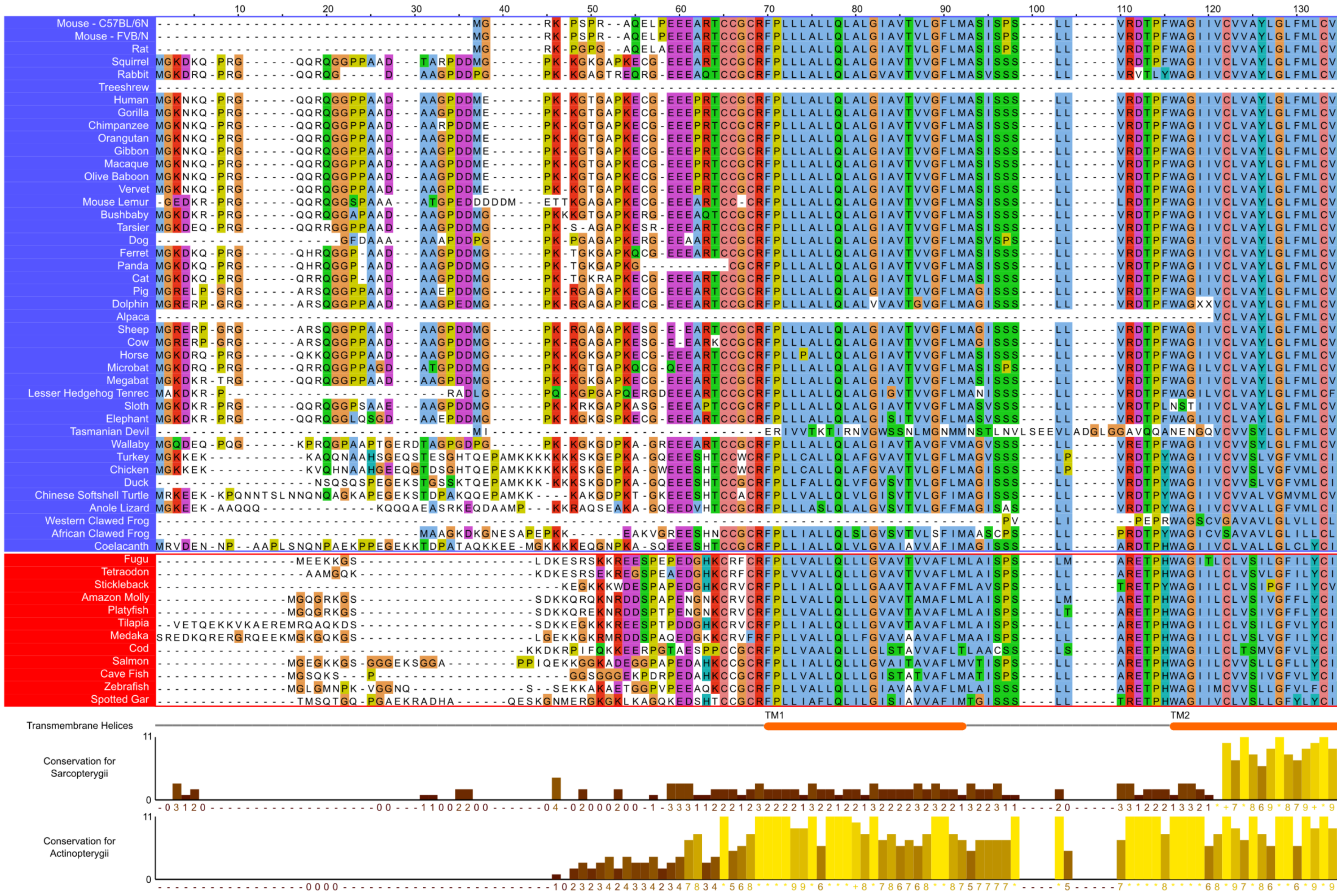
Mouse - C57BL/6N
Mouse - FVB/N
Rat
Squirrel
Rabbit
Treeshrew
Human
Gorilla
Chimpanzee
Orangutan
Gibbon
Macaque
Olive Baboon
Vervet
Mouse Lemur
Bushbaby
Tarsier
Dog
Ferret
Panda
Cat
Pig
Dolphin
Alpaca
Sheep
Cow
Horse
Microbat
Megabat
Lesser Hedgehog Tenrec
Sloth
Elephant
Tasmanian Devil
Wallaby
Turkey
Chicken
Duck
Chinese Softshell Turtle
Anole Lizard
Western Clawed Frog
African Clawed Frog
Coelacanth
Fugu
Tetraodon
Stickleback
Amazon Molly
Platyfish
Tilapia
Medaka
Cod
Salmon
Cave Fish
Zebrafish
Spotted Gar
Transmembrane Helices
TM1
TM2
Conservation for Sarcopterygii
Conservation for Actinopterygii

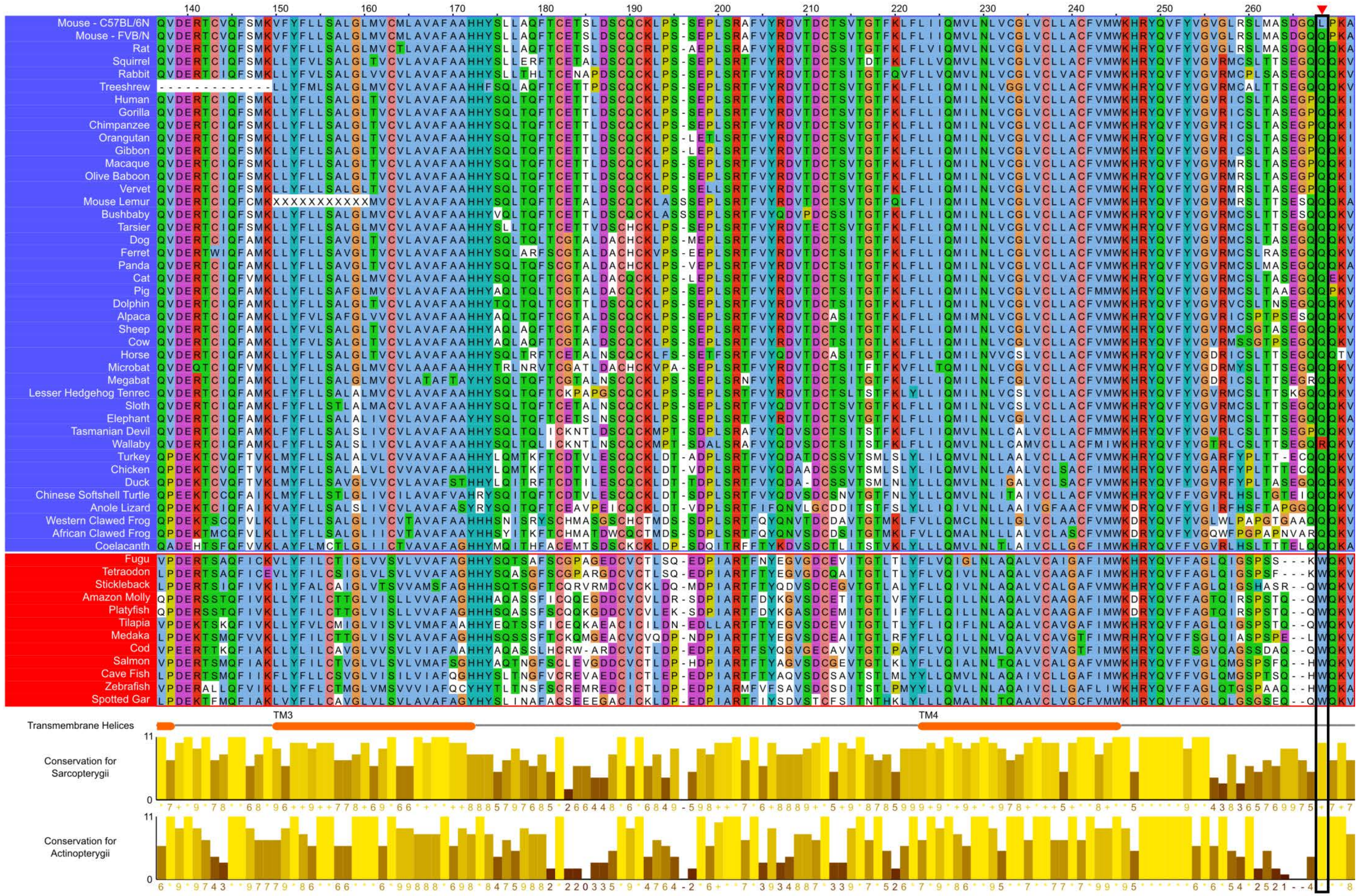
140
150
160
170
180
190
200
210
220
230
240
250
260
Mouse - C57BL/6N
Mouse - FVB/N
Rat
Squirrel
Rabbit
Treeshrew
Human
Gorilla
Chimpanzee
Orangutan
Gibbon
Macaque
Olive Baboon
Vervet
Mouse Lemur
Bushbaby
Tarsier
Dog
Ferret
Panda
Cat
Pig
Dolphin
Alpaca
Sheep
Cow
Horse
Microbat
Megabat
Lesser Hedgehog Tenrec
Sloth
Elephant
Tasmanian Devil
Wallaby
Turkey
Chicken
Duck
Chinese Softshell Turtle
Anole Lizard
Western Clawed Frog
African Clawed Frog
Coelacanth
Fugu
Tetraodon
Stickleback
Amazon Molly
Platyfish
Tilapia
Medaka
Cod
Salmon
Cave Fish
Zebrafish
Spotted Gar
Transmembrane Helices
TM3
TM4
Conservation for Sarcopterygii
Conservation for Actinopterygii
11
0

**Supplementary Figure 1.** Multiple species alignment of SSPN sequences for 37 *Sarcopterygii* (1 lobe-finned fish, 1 amphibian, 2 reptile, 3 bird, and 30 mammal) and 11 *Actinopterygii* (ray-finned fishes). The glutamine at mouse amino acid 213 is nearly invariant in *Sarcopterygii*. C57BL/6 carries a leucine at this position, whereas FVB/N carries the conserved glutamine. The tryptophan at the same position in *Actinopterygii* is invariant. Amino acid conservation is scored separately for the *Sarcopterygii* and *Actinopterygii*. The maximum score, 11, corresponds to perfect conservation of an amino acid across all species.

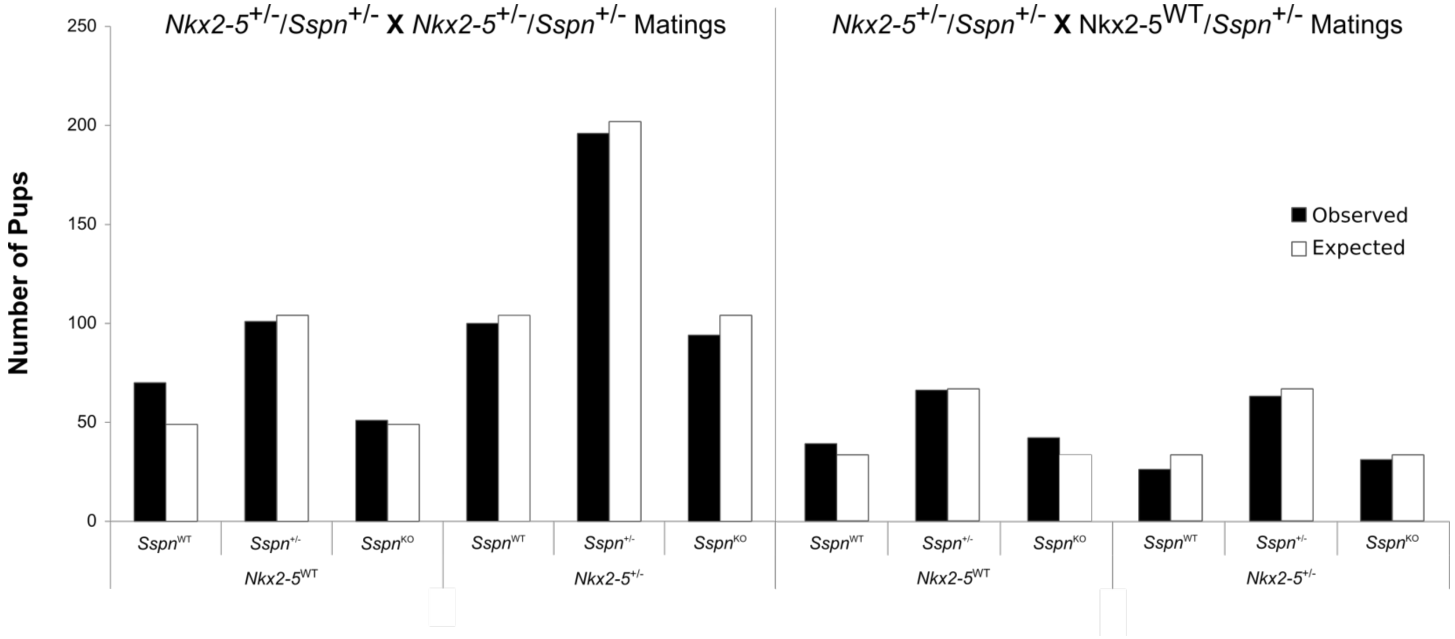


**Supplementary Figure 2.** Crosses of *Nkx2-5*$^{+/-}$/*Sspn*$^{+/-}$ double mutants between themselves or to *Nkx2-5*$^{WT}$/*Sspn*$^{+/-}$ mice yield the expected distribution of genotypes among newborn mouse pups (N = 612 and 267 offspring of each cross, respectively). The *Nkx2-5* knockout is embryonic lethal, so no pups survive to birth.

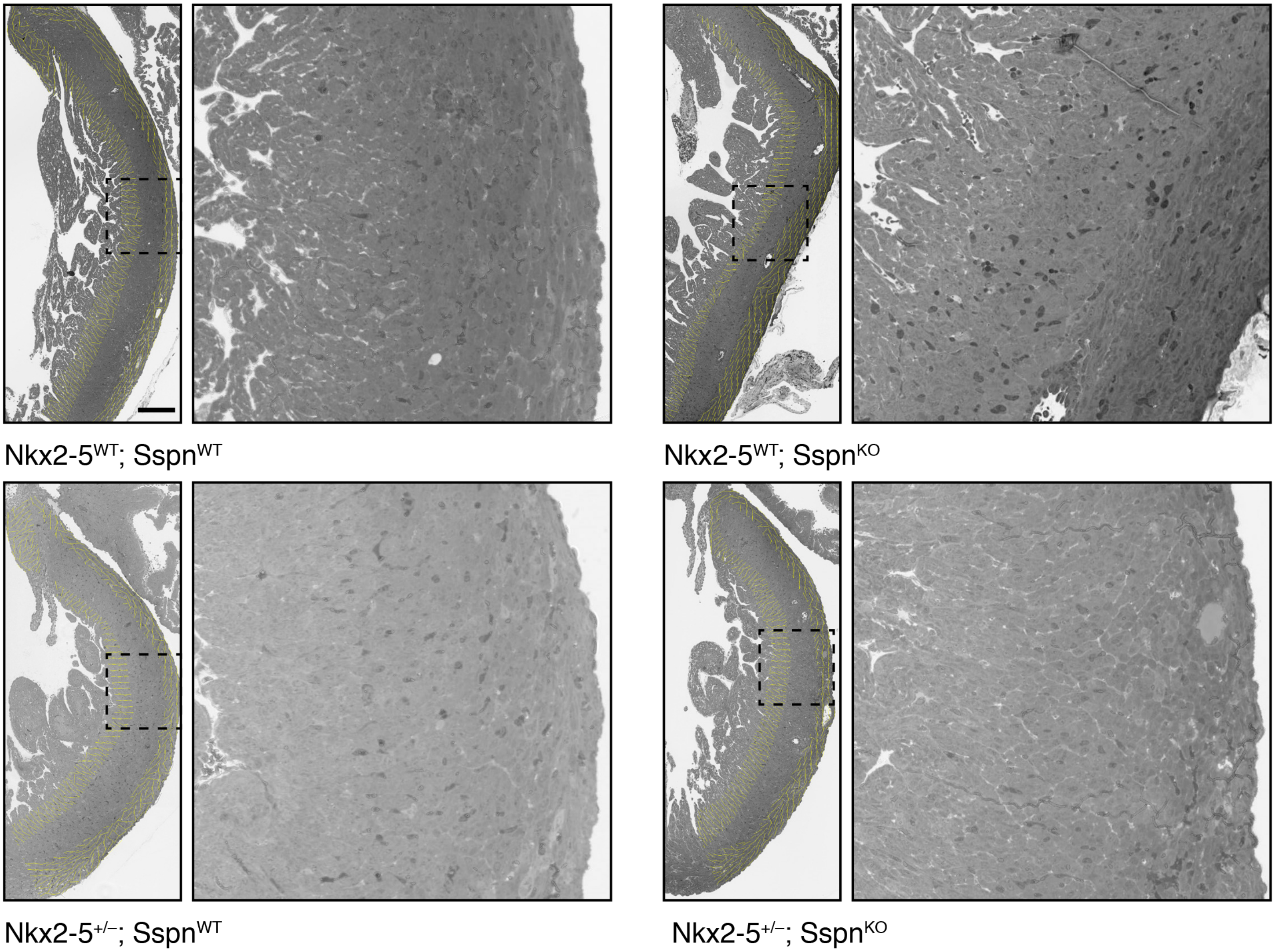


**Supplementary Figure 3.** The orientation of myofibers in the left ventricular free wall is arranged in layers. Subepicardial fibers are oriented predominantly in the plane of section from the apex to the base of the heart, as depicted by the yellow vectors in each panel. Fibers in the middle layer are oriented circumferentially around the left ventricle, i.e., coming out of the plane of the section. The vector orientation of fibers at the base of trabeculae is also shown. The hearts from all genotypic groups show the same myofiber pattern. For clarity, the vectors of circumferential fibers are not shown. Boxed regions are enlarged to show the myofibers. Scale bar, 200 μm.

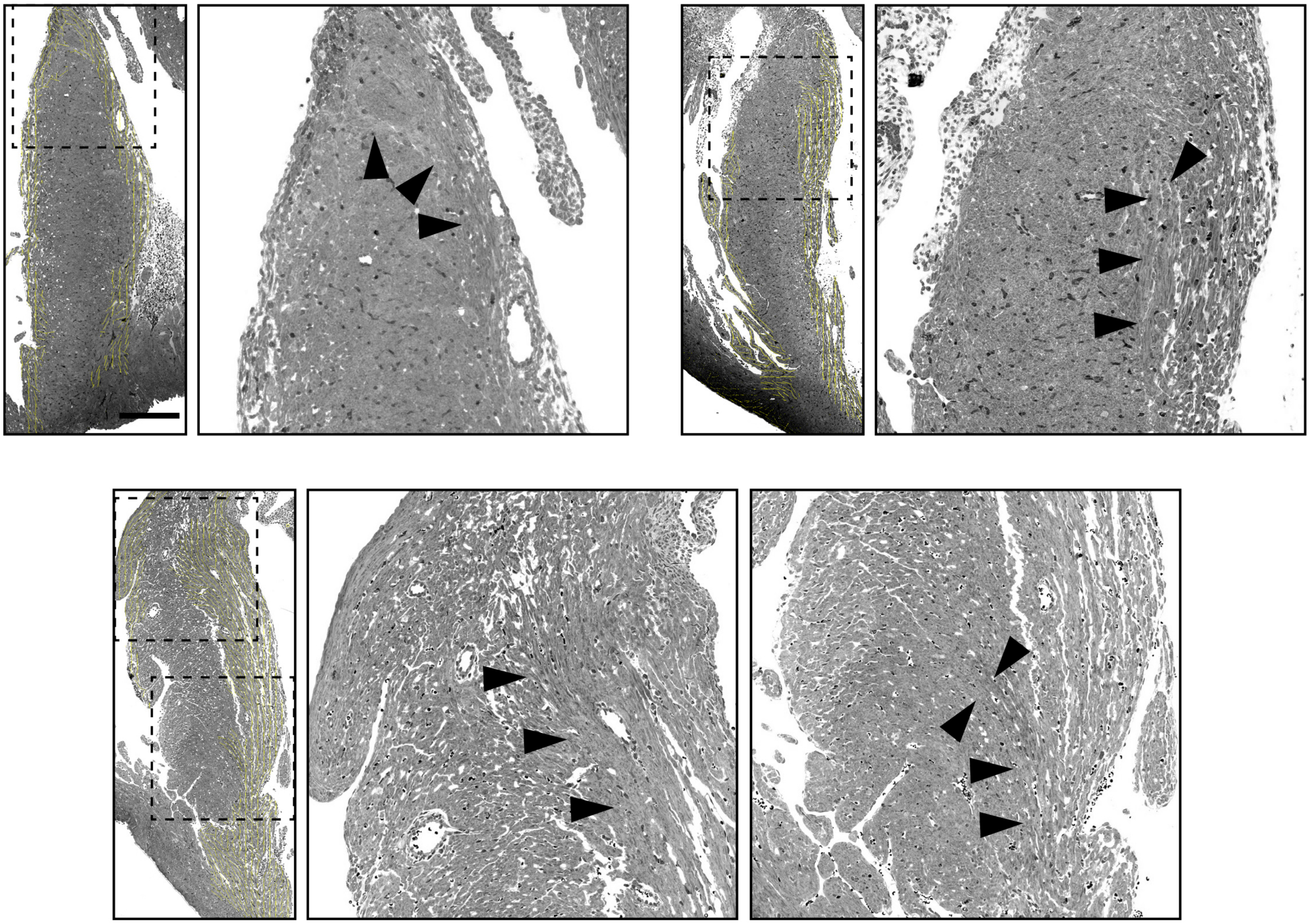

**Supplementary Figure 4.** The deviation of subendocardial fibers along the left side of the ventricular septum was observed in each of the four double mutant *Nkx2-5*$^{+/-}$/*Sspn*$^{KO}$ hearts examined. One of them is shown in Figure 4. The other three hearts are shown here. The yellow vectors indicate myofibers that run parallel to the plane of section. Boxed regions are enlarged to show the malaligned myofibers (arrowheads). Scale bar, 200 μm.
